# Specific Knockdown of Gene Expression in the Mature Rat Pineal Gland: The Cone‐Rod Homeodomain Transcription Factor Regulates Melatonin Synthesis In Vivo

**DOI:** 10.1111/jpi.70177

**Published:** 2026-08-06

**Authors:** Nicole Anne Morrissey, Tenna Bering, Aurea Susana Blancas‐Velázquez, Jakob Albrethsen, Nicolai J. Wewer Albrechtsen, Martin Fredensborg Rath

**Affiliations:** ^1^ Department of Neuroscience, Faculty of Health and Medical Sciences University of Copenhagen Copenhagen Denmark; ^2^ Department of Clinical Biochemistry Copenhagen University Hospital – Bispebjerg and Frederiksberg Copenhagen Denmark; ^3^ Department of Clinical Medicine, Faculty of Health and Medical Sciences University of Copenhagen Copenhagen Denmark

**Keywords:** adeno‐associated virus, homeobox gene, melatonin synthesis, pineal gland, shRNA

## Abstract

Melatonin is synthesised from tryptophan by the sequential action of enzymes that are highly expressed in the pineal gland. Homeobox gene‐encoded transcription factors typically control organ development; however, a set of homeobox genes is strongly expressed in the adult pineal gland. Previous in vitro experiments revealed that knockdown of homeobox genes in rat pinealocyte cultures reduced expression of melatonin‐synthesising enzymes. Until now, it was not possible to determine the impact of homeobox genes on melatonin synthesis in vivo, which is needed to evaluate physiological functions. Using the cone‐rod homeobox (*Crx*) gene as an example, we therefore developed an experimental pipeline to deliver short‐hairpin RNA, via adeno‐associated viral vectors, into the pineal gland of adult rats. This approach enabled us to selectively reduce *Crx* expression in the mature pineal gland, which we confirmed at both the transcript and protein levels. We employed a common approach in pharmacology to correlate *Crx* knockdown with the expression level of the tagged fluorescent reporter, which provided a quantitative basis to define data exclusion/inclusion criteria. Our efforts confirmed that knockdown of *Crx* in vivo reduced the expression of two melatonin‐synthesising enzymes, namely tryptophan hydroxylase 1 and acetylserotonin O‐methyltransferase, consistent with in vitro data. Furthermore, knockdown of pineal *Crx* significantly reduced nighttime plasma melatonin levels. Our work demonstrates a method through which knockdown of target genes in the rat pineal gland can be achieved without the need for transgenic models.

**Abbreviations**
*Aanat*
: aralkylamine N‐acetyltransferase
AAV: adeno‐associated virus
*Actb*
: actin beta
*Asmt*
: acetylserotonin O‐methyltransferase
*Crx*
: cone‐rod homeobox
*egfp*
: enhanced green fluorescent protein
GAPDH: glyceraldehyde 3‐phosphate dehydrogenase
*Ppia*
: peptidylprolyl isomerase A
*Rpl32*
: ribosomal protein L32
RT‐qPCR: real‐time quantitative PCR
shCrx: shRNA targeting *Crx*
shNT: non‐targeting shRNA
shRNA: short hairpin RNA
siRNA: small interfering RNA
*Tph1*
: tryptophan hydroxylase 1
ZT: zeitgeber time

## 1 Introduction

Melatonin is produced at night by the pineal gland and acts as an important endogenous signal for the onset and duration of darkness [1, 2, 3]. The overall circadian melatonin profile is dictated by the suprachiasmatic nucleus of the hypothalamus, via a multisynaptic pathway [1] leading to the nocturnal release of norepinephrine in the gland which activates the local melatonin‐producing machinery [4]. Melatonin is synthesised from tryptophan by the sequential action of enzymes, including tryptophan hydroxylase 1 (TPH1), the norepinephrine‐induced rhythm‐generating aralkylamine N‐acetyltransferase (AANAT), and acetylserotonin O‐methyltransferase (ASMT). Cellular restriction of these enzymes to the melatonin‐producing pinealocytes of the pineal gland appears to depend on local expression of a set of homeobox gene‐encoded transcription factors [5]. Homeobox genes are crucial for developmental processes across tissues. Yet, as a feature of the pineal gland, a number of these transcription factors are still expressed in the adult rat pineal gland and exhibit circadian rhythms [6, 7, 8, 9, 10, 11, 12]. Work in primary cultures of pineal cells suggests that these transcription factors act to control expression of the melatonin‐producing enzymes [9, 12, 13, 14], but evidence from melatonin‐proficient in vivo models is currently missing.

Previous reports based on cell cultures [13, 14] and global knockout mice [15] suggest that the cone‐rod homeobox gene (*Crx*), which is highly expressed in retinal photoreceptors and pinealocytes [6, 16, 17, 18, 19], controls expression of melatonin‐producing enzymes. In the current context, the limited expression domain of *Crx* itself has been used to generate tissue‐specific conditional knockout mouse strains with Cre recombinase expression driven by the *Crx* promoter [20]. However, since *Crx* is also expressed in the retina [16], effects of gene deletions are also detectable in the eye [21]. In addition, the 129/Sv strain, which has been used to generate available *Crx* promoter‐driven Cre recombinase‐expressing mice [22], is melatonin deficient like most other laboratory mouse strains [23], which are therefore not suited for physiological analyses of the photoneuroendocrine circadian system [24]. Genetic targeting in cells expressing *Aanat* provides an alternative method to target the pineal gland in the melatonin‐proficient rat [25, 26]. Still, rodent *Aanat* is expressed in extra‐pineal tissues such as the retina, which hinders pineal‐specific gene knockout. Both the retina and the pineal gland are integral parts of the photoneuroendocrine system [27], and so analysis of the role of individual genes is compromised. As an alternative to these in vivo models, gene knockdown in cultured rat pinealocytes has been widely employed; initially by use of short hairpin RNA (shRNA)‐expressing adenoviruses [13, 28, 29, 30] and later by the use of small interfering RNA (siRNA) [9, 10, 12, 14, 31, 32, 33]. Similar in vitro experiments have been performed in porcine [34] and chick [35] pinealocytes, but attempts have so far been unsuccessful in pineal glands in vivo. While shRNA‐expressing adeno‐associated viruses (AAV) are used to knock down gene expression by injection into the pineal area of newborn rats [36], methods to specifically knock down gene expression in the mature pineal gland currently represent an unsolved challenge preventing progress in this specific scientific field.

With an overall aim to determine the role of *Crx* in regulation of melatonin‐producing enzymes and melatonin synthesis in the developed pineal gland in vivo, we here designed and implemented an AAV‐based method to efficiently knock down gene expression. The results of these efforts, which disclose a role for *Crx* in the regulation of melatonin synthesis in the mature rat pineal gland in vivo, are presented below.

## 2 Materials and Methods

### 2.1 Animals

Male Sprague–Dawley rats (Janvier Labs) were housed under a 12‐h light/dark cycle with ad libitum access to food and water. All animal experiments were conducted at the University of Copenhagen Department of Experimental Medicine and according to the guidelines of EU directive 2010/63/EU and approved by the Danish Council for Animal Experiments (2022‐15‐0201‐01122) and the Faculty of Health and Medical Sciences, University of Copenhagen (P24‐082).

### 2.2 Viral Vectors

AAV vectors for serotype testing were ordered from Addgene (37825). Upon testing several different serotypes, we found that AAV2/9 was the most efficient at infecting the rat pineal gland (Supporting Material, Figure S1). AAV vectors expressing shRNA were produced by VectorBuilder. The sequence to produce shRNA targeting the rat cone‐rod homeobox gene (*Crx*) was already validated in vitro [14]. This sequence was packaged under the U6 promoter and expressed in a plasmid, along with the EGFP reporter under a CMV promoter. This plasmid, pAAV[shRNA]‐EGFP‐U6> (Rat Crx‐targeting shRNA), and the plasmid containing the non‐targeting shRNA control, pAAV[shRNA]‐EGFPU6>Scramble_shRNA, were packaged into AAV2/9 vectors and ultra‐purified by VectorBuilder. The viral preparations were aliquoted upon arrival and stored at −70°C until use.

### 2.3 Intra‐Pineal Injections

**FIGURE 1:**
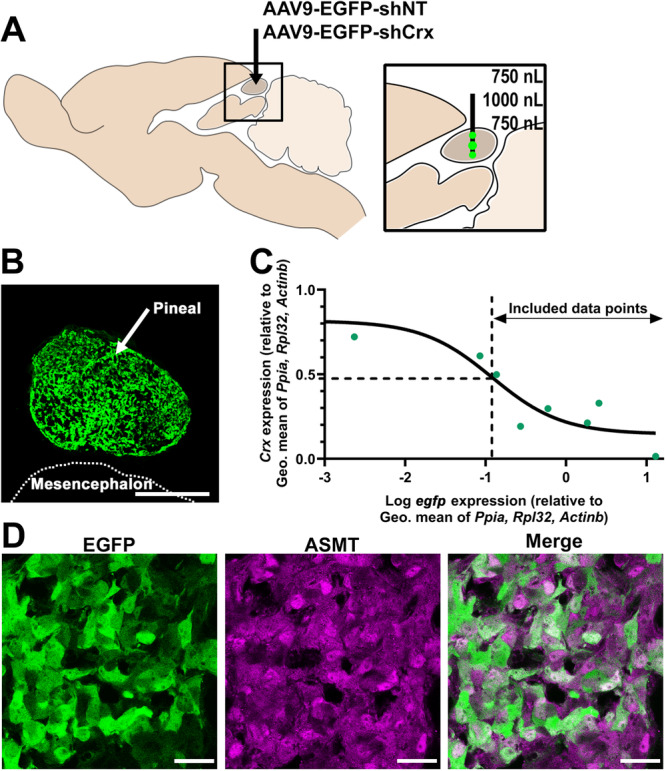
Experiment overview for successful transduction of EGFP expression in the adult rat pineal gland. (A) Schematic for delivery of viral vectors to the rat pineal gland. The enlarged image (right) demonstrates how the injection of 2.5 µL viral vector was delivered over three consecutive infusions at different depths in the pineal. (B) EGFP immunoreactivity in a sagittal section of pineal gland from a rat injected with AAV2/9‐CMV‐EGFP‐U6‐shNT (AAV‐shNT) 14 days before euthanasia. Scale bar, 500 µm. (C) The logarithmic dose‐response curve of *egfp* relative expression, as a proxy for viral transfection, and *Crx* relative expression. The dashed lines indicate the IC50 of viral transfection to reduce *Crx* expression. (D) A representative immunohistochemically processed section from the pineal gland of a rat injected with AAV2/9‐CMV‐EGFP‐U6‐shNT 14 days before euthanasia, showing cells positive for EGFP (green) and ASMT (magenta). Scale bars, 25 µm.

Sprague–Dawley rats (150–250 g) underwent stereotaxic intra‐cranial injections to target the pineal gland. Surgeries were performed under anaesthesia induced and maintained by sevoflurane and with analgesia from preoperative sub‐cutaneous injections of carprofen (5 mg/kg) and bupivacaine (0.25%, 0.1 mL). A single midline incision was made in the skin over bregma to lambda. Pre‐pulled glass micropipettes (BioMedical Instruments) were pre‐filled with Vaseline oil, secured into a 10 µL syringe (Hamilton), and positioned into the microinjector pump head (World Precision Instruments Europe) attached to the stereotaxic frame (Kopf). The tip of the glass micropipette was used to identify the coordinates for bregma, and a single burr hole was drilled in the midline at coordinates −8.3 mm posterior of bregma. PBS or the viral preparations, diluted to 4.13 × 10^12^ genome copies per mL in sterile PBS, were infused at a rate of 200 nL/min over three positions: 750 nL at −2.5 mm, 1000 nL at −2.3 mm, and 750 nL at −2.1 mm from the dura (Figure 1A). The micropipette was left in place for 2 min after each infusion and withdrawn gradually over 3 min after the final infusion. The incision site was sutured, and the animals were monitored for 5 days post‐surgery, with sub‐cutaneous injections of carprofen (5 mg/kg) given for the first 2 postoperative days.

### 2.4 Euthanasia and Tissue Collection

The rats were left to recover for 14–18 days following intra‐pineal injection of the viral preparations and to enable efficient infection of the AAVs and transduction of the genetic material. Prolonged infection periods (20–27 days) reduced pineal *Crx* expression even in rats injected with AAVs encoding the non‐targeting shRNA control (Supporting Material, Figure S2), so animals were euthanised no later than 18 days post‐injection of the viral preparations. For RNA, protein extraction, and melatonin measurements, the rats were euthanised at zeitgeber time 18 (ZT18) by exposure to a rising concentration of carbon dioxide followed by decapitation. Trunk blood was collected and processed as previously described [37]. The pineal glands were frozen on dry ice and stored at −70°C until processing. For immunohistochemical processing, animals were perfusion fixed as previously described [38].

### 2.5 RNA Extraction, cDNA Synthesis, and RT‐qPCR

Whole pineal glands were homogenised in TRIzol (Invitrogen) on ice and RNA extracted using the TRIzol/chloroform method. The RNA was suspended in DNA/RNA‐free water and treated with DNase I (Invitrogen) according to the manufacturer's protocol. cDNA was synthesised using Superscript III reverse transcriptase (Invitrogen). Real‐time PCR was run on a Lightcycler 96 RT‐qPCR machine (Roche), with primer sequences specific to the target of interest (Table 1) diluted to 0.5 µM in FastStart Essential DNA Green Master (Roche). Transcript levels were calculated by the standard curve method, using serial dilutions of plasmids containing the target sequence of the primers. These were normalised to the geometric mean of actin beta (*Actb*), peptidylprolyl isomerase A (*Ppia*), and ribosomal protein L32 (*Rpl32*) transcript levels.

**TABLE 1:** Primer sets used for RT‐qPCR.

Transcript	Genbank ID	Position	Forward primer (5′–3′)	Reverse primer (5′–3′)
*Aanat*	NM_012818.2	525–682	TGCTGTGGCGATACCTTCACCA	CAGCTCAGTGAAGGTGAGAGAT
*Actb*	NM_031144.3	026–184	ACAACCTTCTTGCAGCTCCTC	CCACGATGGAGGGGAAGAC
*Asmt*	NM_144759.2	696–860	GCAAGACCCAGTGTGAGGTT	CAGTAGTGCACCACCTGGC
*Crx*	NM_021855.2	076–204	ATGCACCAGGCTGTCCCATA	TGCATACACATCCGGGTACTG
*egfp*	NW_013564809.1	457–624	ATCATGGCCGACAAGCAGAA	TCTCGTTGGGGTCTTTGCTC
*Ppia*	NM_017101.1	383–450	TCTGCACTGCCAAGACTGAG	CATGCCTTCTTTCACCTTCC
*Rpl32*	NM_013226.3	072–196	GGTGAAGCCCAAGATCGTCA	ATCTTCTCCGCACCCTGTTG
*Tph1*	NM_001100634.3	702–814	CAGAAACCTTCCTCTGCTCTCA	CAGGACGGATGGAAAACCCT

### 2.6 Fluorescent Immunohistochemistry

Sagittal 12 µm cryostat sections were processed by fluorescent immunohistochemistry as previously described [14]. In brief, sections were blocked in either 5% donkey or 5% goat serum in PBS before incubation with the antibodies of choice to detect ASMT, CRX, and EGFP protein (Table 2). In the case of CRX immunohistochemistry, sections underwent antigen retrieval by incubation in Dako Target Retrieval Solution Citrate pH 6 (Agilent) at 80°C for 1.5 h before incubation with primary antibody. Sections were incubated with primary antibody overnight at 4°C, and in secondary antibody for 2.75 h at room temperature. The primary antibody targeting EGFP was added to the solution containing the secondary antibodies. Sections were counter‐stained with DAPI before being coverslipped with fluorescence mounting medium (Dako) and stored at 4°C. Sections were imaged by confocal microscopy on an LSM 710 microscope using Zen Black software (Zeiss), with the settings unchanged between sections. DAPI staining was used for orientation purposes. Images for figures were prepared using ImageJ FIJI (V1.54, NIH), with brightness and contrast adjusted equally.

**TABLE 2:** Antibodies used for immunohistochemistry.

Primary antibody			Secondary antibody		
Name	Source	Dilution	Name	Vendor	Dilution
Rabbit anti‐ASMT	David Klein, 8091 [42]	1:200	Goat anti‐rabbit, Alexa Fluor 568	Invitrogen, A11011	1:400
Rabbit anti‐CRX	NeoMPS, 448704 [14]	1:400			
GFP‐Booster Alexa Fluor 488	ChromoTek, gb2AF488	1:1000			

### 2.7 Protein Extraction and Western Blot

Whole pineal glands, 1 gland per tube, were homogenised and protein extracted as previously described [14]. In brief, glands were homogenised in 50 µL 2x Laemmli buffer, boiled, and then centrifuged. The amount of protein extracted was measured in 1 µL of supernatant, in triplicate, by Pierce BCA Protein Assay kit (VWR) according to the manufacturer's instructions. Fifty micrograms of protein per sample was boiled in 2x Laemmli buffer with 10% β‐mercaptoethanol and then added to the wells of a 4%–12% NuPAGE Bis‐Tris gel (Invitrogen), which was assembled in an XCell Surelock Mini‐Cell system (Invitrogen). The gel was run at 70 V for 25 min followed by 100 V for 2 h. The separated proteins were transferred to a nitrocellulose membrane and then blocked in 2% semi‐skimmed milk powder dissolved in PBS. The membrane was probed with primary antibodies overnight at 4°C, except GAPDH which was incubated for 1 h at room temperature, and with secondary antibodies for 1 h at room temperature (Table 3). The membrane was imaged using Sapphire Biomolecular Imager (Azure Biosystems), and the bands were quantified using ImageJ FIJI.

**TABLE 3:** Antibodies used for Western blot.

Primary antibody			Secondary antibody		
Name	Source	Dilution	Name	Vendor	Dilution
Rabbit anti‐ASMT	David Klein, 8091 [42]	1:500	Goat anti‐rabbit, Alexa Fluor 680	Invitrogen, A‐21076	1:1000
Rabbit anti‐CRX	NeoMPS, 448704 [14]	1:500			
Rabbit anti‐EGFP	Invitrogen, A‐11122	1:1000			
GAPDH DyLight 800	Invitrogen, MA5‐15738‐D800	1:1500			
Mouse anti‐TPH1	Millipore, MAB5278	1:10 000	Goat anti‐mouse, Alexa Fluor 680	Invitrogen, A‐21057	1:1000

### 2.8 Melatonin Measurements

Plasma melatonin was determined by Liquid Chromatography Tandem Mass Spectrometry following a previously published protocol [39].

### 2.9 Statistical Analysis

Data were analysed and graphs were produced in Prism software (V10.2, GraphPad). Statistical analysis between two groups was performed using Student's unpaired *t*‐test; unless the standard deviation between the two groups was unequal, in which case Welch's unpaired *t*‐test was used. Pearson's correlation coefficient was used to analyse correlation. A *p*‐value of < 0.05 was considered to represent statistical significance. To determine inclusion/exclusion criteria for successful AAV injections and transfections, relative *Crx* expression levels were plotted against the corresponding log of relative *egfp* expression to give a dose‐response inhibition curve. A three‐parameter inhibitor‐response curve was fitted, which gave an IC50 value of 0.121 relative Egfp expression and was used as the lower limit of inclusion in the RT‐qPCR analysis. Data inclusion criteria for Western blots were based on whether a band corresponding to the expected size of EGFP was detected in the samples. Figures were produced using Photoshop Elements (V2024, Adobe).

## 3 Results

### 3.1 The Adult Rat Pineal Gland Can Be Transduced Through Targeted Injection of Adeno‐Associated Viral Vectors to Knock Down Gene Expression

We here demonstrate that shRNA can be introduced to the adult rat pineal gland in vivo through stereotaxic delivery of AAV vectors. We introduced shRNA that targets the *Crx* transcript (shCrx), or non‐targeting shRNA (shNT) in control animals, alongside an EGFP reporter. We infused the viral preparations into the pineal gland over three different coordinates in the *z*‐axis to achieve sufficient viral spread (Figure 1A). To reduce the impact of the immune response in the pineal gland to the AAVs, while still maintaining efficient transduction of genetic material, we diluted the viral preparations before injection and increased the injection volume. This resulted in efficient transduction of the EGFP reporter protein (Figure 1B). We found that *egfp* relative expression, as a proxy for successful AAV‐shCrx transfection, could predict the relative expression of *Crx* vs. PBS‐injected controls (data points not shown for clarity) with a common pharmacological model (Figure 1C, goodness of fit: *R* ^2^ = 0.84, deviation from model: *p* = 0.87). The relative expression level of *egfp* that corresponded to a 50% reduction in *Crx* expression was 0.12, which we then used to determine inclusion criteria for qPCR analysis of samples with sufficient transfection with the AAVs. We used immunohistochemistry in pineal‐containing brain sections to identify the cells infected by the virus. The infected cells included melatonin‐synthesising pinealocytes, as observed by co‐localisation of EGFP with ASMT immunoreactivity (Figure 1D). *egfp* expression was undetectable in the eyes (Cq‐values > 34), and retinal *Crx* expression did not differ between experimental groups (Supporting Material, Figure S3).

### 3.2 Pineal Expression of *Crx* Is Modulated by Delivery of Short‐Hairpin RNA in Adeno‐Associated Viral Vectors in Rats In Vivo

**FIGURE 2:**
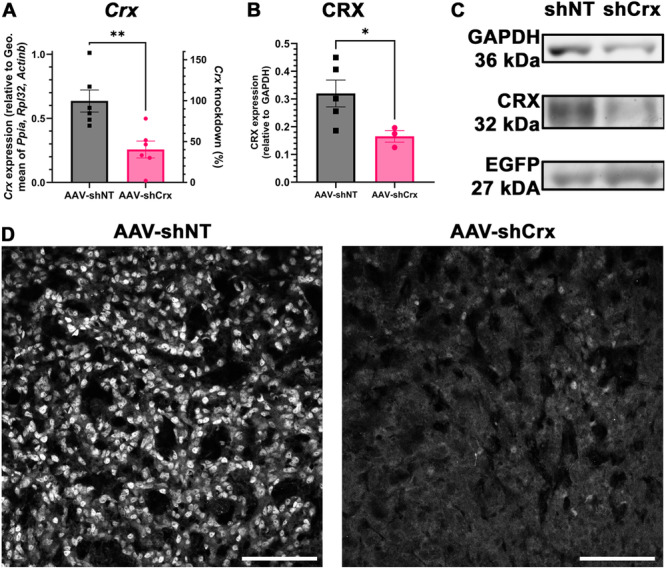
Rats injected with AAV‐shCrx in the pineal gland exhibit reduced levels of *Crx* mRNA transcripts and CRX protein. (A) *Crx* mRNA levels in the pineal gland of rats injected with either AAV‐shNT or AAV‐shCrx. The percentage of *Crx* knockdown (plotted on the *y*‐axis on the right‐hand side) is calculated using the average *Crx* level in the AAV‐shNT‐injected controls as 100%. (B) CRX protein expression in the pineal gland of rats injected with either AAV‐shNT or AAV‐shCrx. (C) A cropped image of representative bands from the western blot (Supporting Material, Figure S4), containing protein extracted from the pineal gland of rats injected with either AAV‐shNT or AAV‐shCrx. The bands correspond to GAPDH (36 kDa) as a loading control, CRX (32 kDa), and EGFP (27 kDa) as a positive control for the success of the injections. (D) CRX‐positive immunoreactivity in representative sagittal sections of the pineal gland from rats injected with AAV‐shNT and AAV‐shCrx 14 days before euthanasia. Scale bars, 100 µm. Data are presented as mean ± SEM. Data were analysed by Student's *t*‐test (A) and Welch's *t*‐test (B). *p < 0.05; **p < 0.01.

The sequence of the shRNA targeting *Crx* has previously been validated to knockdown expression in pinealocyte‐containing cell culture by transfection of *Crx*‐targeting siRNA [14]. We aimed to replicate those results in an in vivo model. Delivery of AAV‐shCrx to the rat pineal gland resulted in a strong and significant reduction of pineal *Crx* mRNA compared to the pineal glands of rats receiving AAV‐shNT (Figure 2A, *p* = 0.006, Student's *t*‐test). This corresponded to an average reduction of 60% (Figure 2A). CRX protein levels were also lower in rats that received AAV‐shCrx than in rats injected with AAV‐shNT (Figure 2B, *p* = 0.03, Welch's *t*‐test), as quantified by Western blot (Figure 2C; Supporting Material, Figure S4). This finding is supported by immunohistochemical staining of brain sections containing the pineal gland, where CRX‐positive immunoreactivity is lower in AAV‐shCrx‐injected rats than in controls (Figure 2D).

### 3.3 Knockdown of *Crx* in the Rat Pineal Gland In Vivo Leads to a Reduction in the Expression of Melatonin‐Synthesising Enzymes and Subsequent Melatonin Synthesis

**FIGURE 3:**
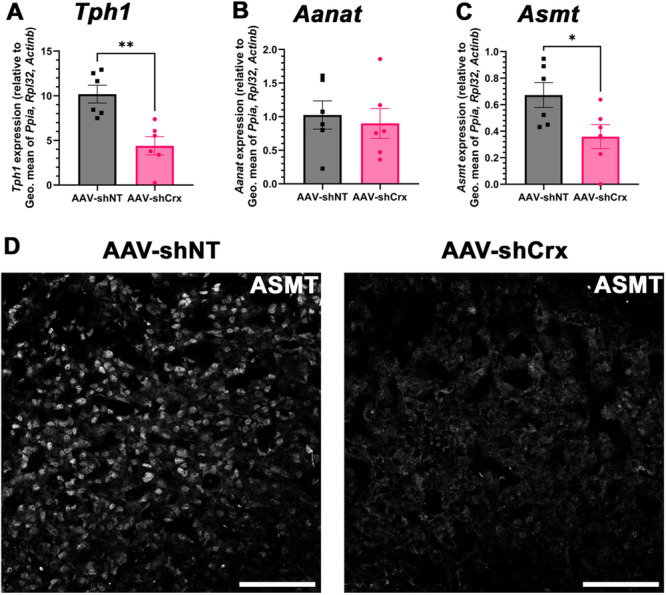
Knockdown of *Crx* expression in the rat pineal gland leads to a reduction in the levels of products of genes encoding the melatonin‐synthesising enzymes. (A) *Tph1*, (B) *Aanat*, and (C) *Asmt* mRNA transcript levels in the pineal glands of rats injected with either AAV‐shNT or AAV‐shCrx. (D) ASMT‐positive immunoreactivity in representative sagittal sections of the pineal gland from rats injected with AAV‐shNT and AAV‐shCrx 15 days before euthanasia. Scale bars, 100 µm. Data are presented as mean ± SEM. Data were analysed by Student's *t*‐test. *p < 0.05; **p < 0.01.

Following successful knockdown of *Crx*, both at the mRNA and at the protein level, we investigated whether this influenced expression of the enzymes in the melatonin synthesis pathway. Injection of AAV‐shCrx to the pineal gland of adult rats resulted in lower levels of *Tph1* (Figure 3A, *p* = 0.002, Student's *t*‐test) and *Asmt* (Figure 3C, *p* = 0.037, Student's *t*‐test) mRNA transcripts than in rats that received the AAV‐shNT control injection. However, there was no effect on *Aanat* mRNA levels (Figure 3B, *p* = 0.69, Student's *t*‐test), which is the rate‐limiting enzyme for melatonin synthesis during daytime. Similar results with reduced pineal expression levels of *Tph1* and *Asmt* following injection of AAV‐shCrx but no effect on *Aanat* were obtained in a second independent series of experiments (Supporting Material, Figure S5). Positive immunoreactivity for ASMT, in brain sections containing the pineal gland, indicated that knockdown of *Crx* by injection of AAV‐shCrx impacted ASMT expression at the protein level compared to AAV‐shNT injected rats (Figure 3D).

**FIGURE 4:**
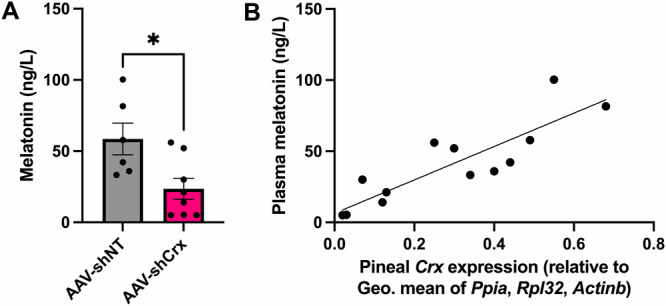
Knockdown of *Crx* expression in the rat pineal gland leads to a reduction in nighttime blood melatonin levels. (A) Melatonin levels in the plasma of rats injected with either AAV‐shNT or AAV‐shCrx in the pineal gland. Data are presented as mean ± SEM and analysed by Student's *t*‐test. *p < 0.05; **. (B) *Crx* expression in the pineal gland plotted against plasma melatonin levels. Line depicts a simple linear regression. Rats from both experimental groups are included. RT‐qPCR data on the pineal glands of the rats used specifically in this figure are available in Supporting Material, Figure S5.

To determine if knockdown of *Crx* would influence pineal melatonin synthesis, nighttime plasma melatonin was measured (Figure 4). Injection of AAV‐shCrx to the pineal gland resulted in lower levels of melatonin detected at ZT18 (Figure 4A, *p* = 0.0181, Student's *t*‐test). In addition, a positive correlation between pineal *Crx* expression and plasma melatonin was detectable (Figure 4B, *r* = 0.88, *p* < 0.0001, Pearson's correlation coefficient analysis).

## 4 Discussion

The modification of gene expression is a useful tool to identify the function of the gene, particularly so when combined with spatial selectivity. This is easily achieved in mouse models compared to rats, due to the number of transgenic lines and viral tools that manipulate gene expression with high spatial and temporal specificity. However, mouse models are not well‐suited for studying the synthesis of melatonin since most laboratory mouse models are melatonin deficient [23]. Here, we translated a commonly used approach to manipulate gene expression in mice to target gene expression in the rat pineal gland instead. Using *Crx* as an example, we developed a rat knockdown model to determine its function in the synthesis of melatonin. While this was previously achieved in pinealocytes in vitro [13, 14], our approach here considers the entire physiology of the rat. *Crx* knockout mouse models exist but, due to the crucial role of *Crx* in development, germline deletion results in abnormal development of the visual system [15]. Therefore, an organ‐specific postnatal knockout model of *Crx* deletion avoids developmental deficiencies in the pineal gland and other organ systems.

In addition to the development of a rat knockdown model using AAVs, we adopted a common pharmacological approach to determine the relationship between *egfp* expression, as a proxy for successful AAV infection, and efficacy of *Crx* knockdown. Typically, the success of AAV injections is determined by visual expression of the fluorescent protein tag by fluorescent microscopy. Since we wished to quantify expression of *Crx* and melatonin‐synthesising enzymes by qPCR, we could not also validate the success of injections visually. The approach applied here to determine data inclusion criteria not only enables us to quantitatively assess the success of injection, but it would also reduce bias. Here, we used a model based on the half‐maximal inhibitory concentration (IC50) model, which traditionally measures the potency of an agent to inhibit a receptor or biological function, to which our data fitted strongly. Therefore, we took the *egfp* expression level that was equivalent to a 50% reduction in *Crx* expression as our minimum inclusion criteria.

We found that knockdown of *Crx* downregulated expression of two key enzymes in the melatonin synthesis pathway, *Tph1* and *Asmt* [4]. This agrees with previous data on cultured pinealocytes in vitro [14]. However, we saw no effect of *Crx* knockdown on expression levels of *Aanat* as observed in vitro [13, 14]. The likely explanation is the differences between the variability of the environment of the in vitro and in vivo models. For instance, the rhythmic expression of *Aanat* is regulated by norepinephrine [4], which we did not control in vivo. Comparison of previous in vivo and in vitro experiments suggests that *Aanat* transcript levels are more stable in cultured cells of the pineal gland [40]. That knockdown of *Crx* had no direct effect on *Aanat* expression was confirmed in a separate series of experiments. On the other hand, decreasing the expression of *Tph1* and *Asmt* by *Crx* knockdown was sufficient to significantly reduce nighttime melatonin levels in vivo, which supports previous work suggesting that during nighttime ASMT is the rate‐limiting enzyme in melatonin synthesis of the rat pineal gland [41]. ASMT is predominantly expressed in a subpopulation of pinealocytes named α‐pinealocytes [42, 43], which also express higher levels of *Crx* as compared to β‐pinealocytes [43]. Although the present study did not include single‐cell analyses, the clear correlation between pineal *Crx* expression and blood melatonin levels supports the notion that melatonin is mainly produced by *Crx* and *Asmt* expressing α‐pinealocytes.

The methodology presented here opens new paths of research in molecular biology of the melatonin‐proficient pineal gland in vivo and will enable examination of the effects of pineal‐specific gene knockdown on the circadian physiology of the rat. Knockdown of *Crx* expression alongside expression of other homeobox genes [5] will provide further knowledge into how the homeobox transcription factors interact to regulate melatonin synthesis in the pineal gland.

## Author Contributions

Martin Fredensborg Rath conceived the study. Nicole Anne Morrissey and Martin Fredensborg Rath designed experiments. Aurea Susana Blancas‐Velázquez performed preliminary experiments. Nicole Anne Morrissey, Tenna Bering, and Jakob Albrethsen performed experiments. Nicolai J. Wewer Albrechtsen and Martin Fredensborg Rath supervised experiments. Nicole Anne Morrissey, Tenna Bering, and Martin Fredensborg Rath analysed results. Nicole Anne Morrissey and Martin Fredensborg Rath prepared the manuscript. All authors approved the final manuscript.

## Conflicts of Interest

The authors declare no conflicts of interest.

## Supporting information


Supporting File

## Data Availability

The data that support the findings of this study are available from the corresponding author upon reasonable request.
