# Supplementary material for: Specific Knockdown of Gene Expression in the Mature Rat Pineal Gland: The Cone‐Rod Homeodomain Transcription Factor Regulates Melatonin Synthesis In Vivo
Source: J Pineal Res. 2026 Aug 6;78(5):e70177. doi: 10.1111/jpi.70177 (PMC13444204; doi:10.1111/jpi.70177)
Supplement: Supplementary file 1 [file JPI-78-e70177-s001.pdf]

Supplementary material

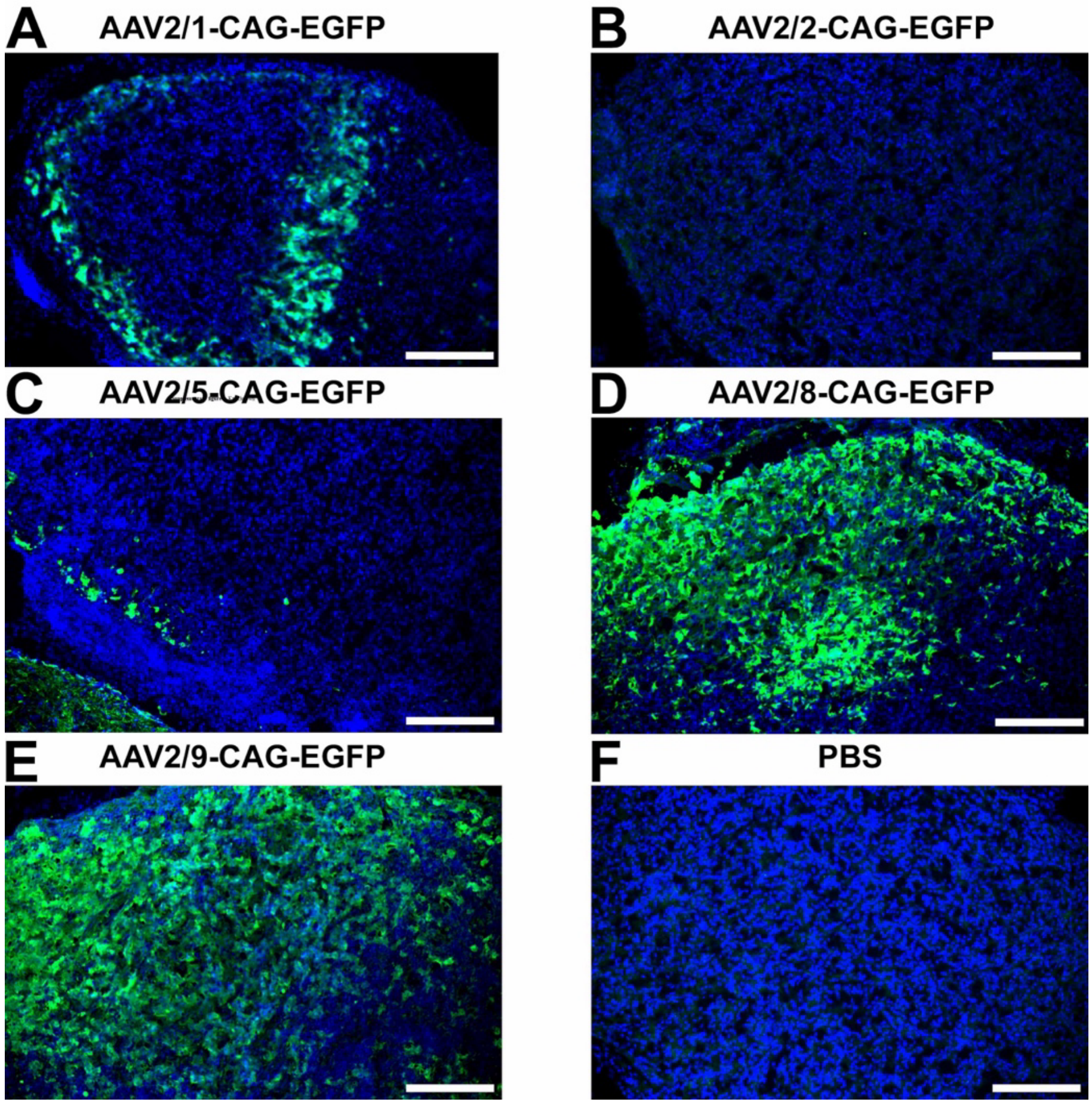

Figure S1. Distribution of AAV infection in the rat pineal gland, using EGFP expression as a proxy. To determine which AAV serotype infected the pineal gland the most efficiently, a selection of different serotypes were tested: (A) AAV2/1, (B) AAV2/2, (C) AAV2/5, (D) AAV2/8, (E) AAV2/9, and a PBS-injected pineal (F) as a negative control. Sections were photographed in an Axioplan 2 epifluorescence microscope (Carl Zeiss Microscopy). Scale bars, 200 μm.

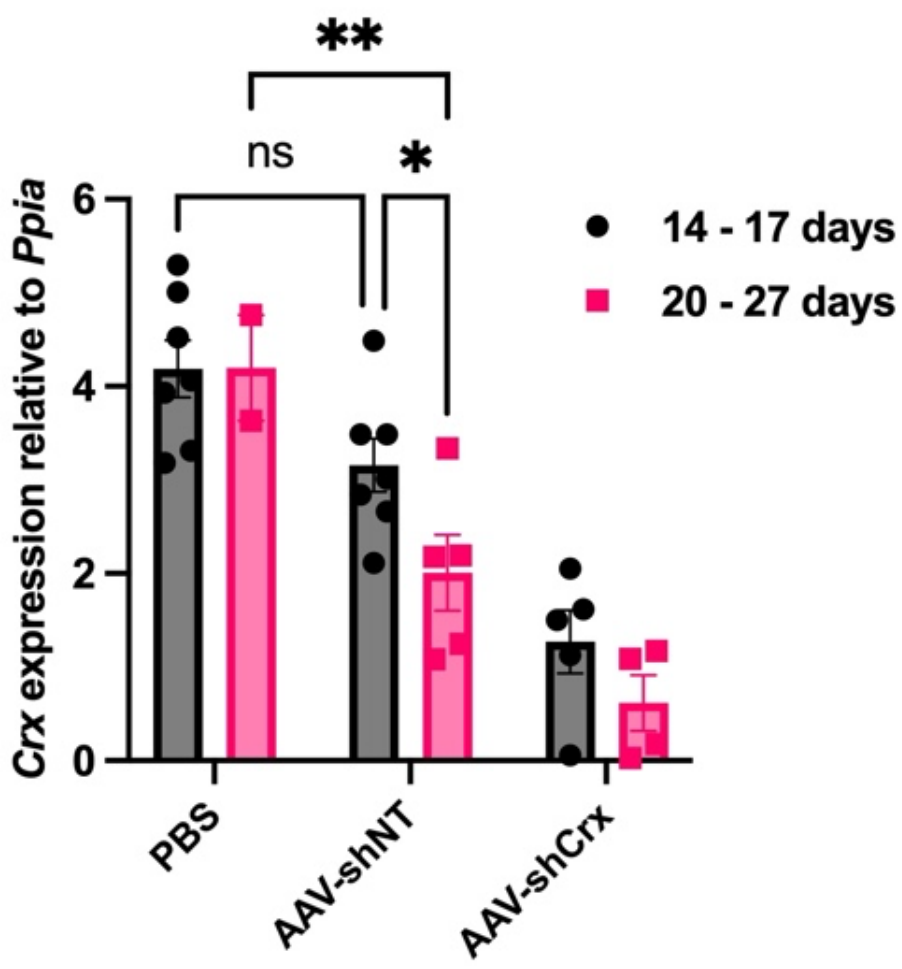

Figure S2. *Crx* expression in the pineal gland changes over time following AAV injections. While pineal *Crx* expression levels do not differ between PBS-injected controls and AAV-shNT-injected animals after 14-17 days, a significant reduction was detectable after 20-27 days. qPCR data were analysed by two-way ANOVA followed by Tukey's multiple comparison test. *, $P < 0.05$; **, $P < 0.01$.

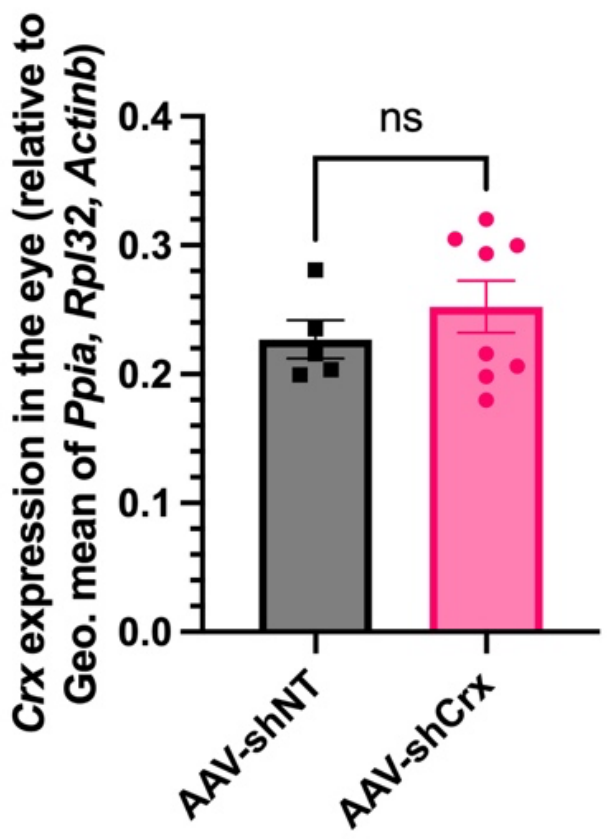

Figure S3. Expression of *Crx* is not affected in the eye following injection of AAV-shCrx in the pineal gland. In the same animals a significant reduction in pineal *Crx* was detectable (Fig. S5). Whole eyes were analysed. Data are presented as mean ± SEM and analysed by Student's t-test. This finding is in accord with *egfp* transcripts being undetectable in the eyes of animals injected with AAV-shCrx and AAV-shNT in the pineal gland, thus suggesting that the virus does not spread to the eyes.

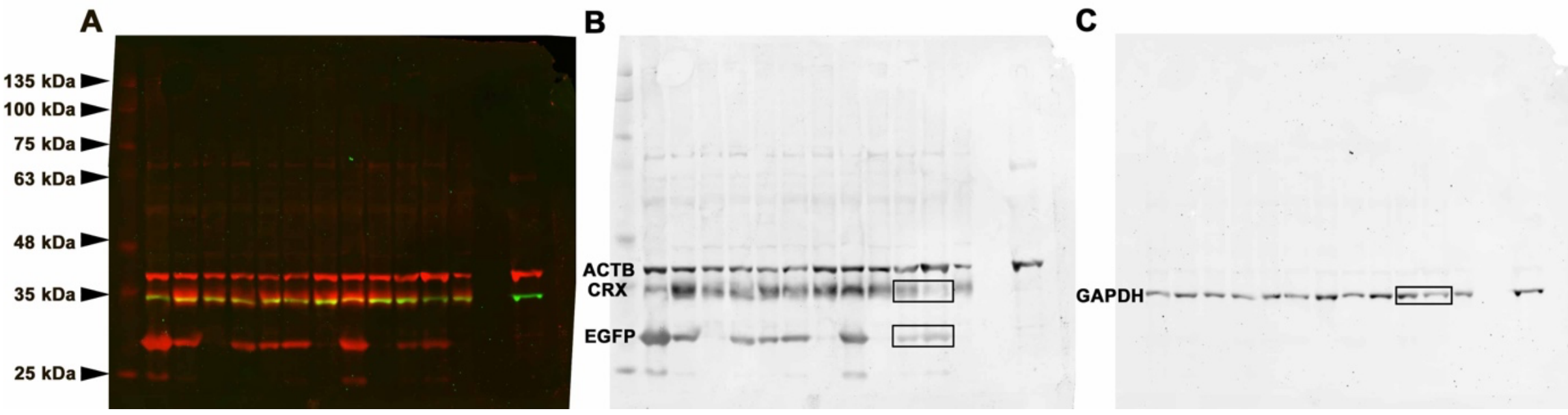

Figure S4. The whole western blot used for analysis in Figure 2A-C. A) The western blot was scanned using two different lasers. B) Inverted grayscale image of the signal in the 685 nm channel. C) Inverted grayscale image of the signal in the 784 nm channel. The outlined boxes signify from where the cropped representative bands were taken for CRX (32 kDa), EGFP (27 kDa) and GAPDH (36 kDa). ACTB (42 kDa) was included as an additional loading control.

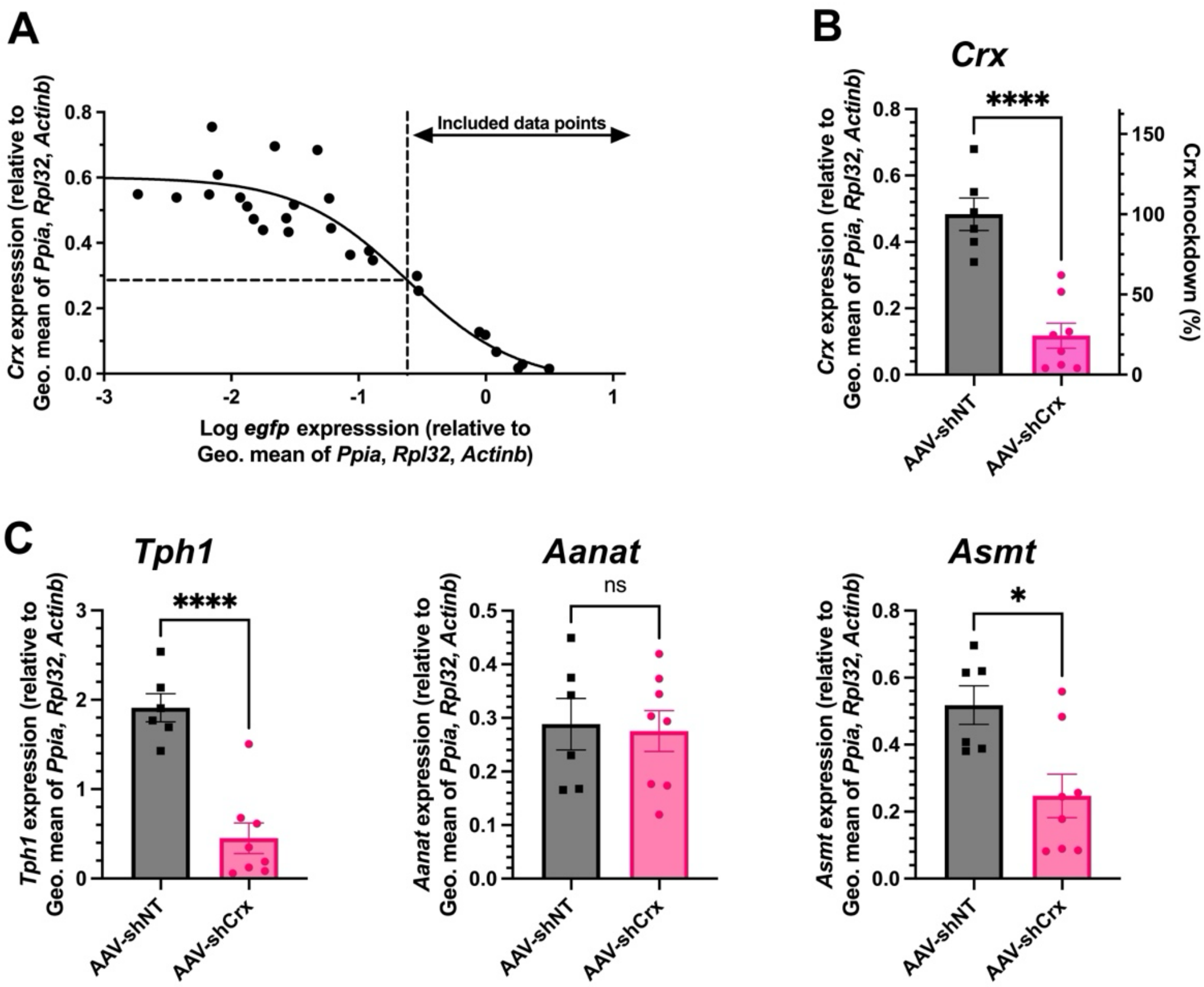

Figure S5. Pineal gene expression data on experimental series specifically used for plasma melatonin measurements. (A) The logarithmic dose-response curve of *egfp* relative expression and *Crx* relative expression. The dashed lines indicate the IC50 of viral transfection to reduce *Crx* expression. (B) *Crx* mRNA levels in the pineal gland of rats injected with either AAV-shNT or AAV-shCrx with percentage of *Crx* knockdown plotted on the right y axis. (C) Expression levels of genes encoding the melatonin-synthesising enzymes in the pineal glands of rats injected with either AAV-shNT or AAV-shCrx. Data are presented as mean ± SEM and analysed by Student's t-test. *, $P < 0.05$; ****, $P < 0.0001$.
